# A Novel and Efficient High-Yield Method for Preparing Bacterial Ghosts

**DOI:** 10.3390/toxins13060420

**Published:** 2021-06-13

**Authors:** Yi Ma, Liu Cui, Meng Wang, Qiuli Sun, Kaisheng Liu, Jufang Wang

**Affiliations:** 1 School of Biology and Biological Engineering, South China University of Technology, Guangzhou 510006, China; bimayikobe@scut.edu.cn (Y.M.); bicuiliu888@mail.scut.edu.cn (L.C.); biandyvictorymeng@mail.scut.edu.cn (M.W.); msqiulisun2016@mail.scut.edu.cn (Q.S.); 2 Guangdong Provincial Key Laboratory of Fermentation and Enzyme Engineering, South China University of Technology, Guangzhou 510006, China; 3 Shenzhen People’s Hospital (The Second Clinical Medical College, Jinan University; The First Affiliated Hospital, Southern University of Science and Technology), Shenzhen 518020, China

**Keywords:** bacterial ghosts, ΦX174, lysis efficiency, pLysS

## Abstract

Bacterial ghosts (BGs) are empty cell envelopes possessing native extracellular structures without a cytoplasm and genetic materials. BGs are proposed to have significant prospects in biomedical research as vaccines or delivery carriers. The applications of BGs are often limited by inefficient bacterial lysis and a low yield. To solve these problems, we compared the lysis efficiency of the wild-type protein E (E_W_) from phage ΦX174 and the screened mutant protein E (E_M_) in the *Escherichia coli* BL21(DE3) strain. The results show that the lysis efficiency mediated by protein E_M_ was improved. The implementation of the pLysS plasmid allowed nearly 100% lysis efficiency, with a high initial cell density as high as OD_600_ = 2.0, which was higher compared to the commonly used BG preparation method. The results of Western blot analysis and immunofluorescence indicate that the expression level of protein E_M_ was significantly higher than that of the non-pLysS plasmid. High-quality BGs were observed by SEM and TEM. To verify the applicability of this method in other bacteria, the T7 RNA polymerase expression system was successfully constructed in *Salmonella enterica* (*S. Enterica*, SE). A pET vector containing E_M_ and pLysS were introduced to obtain high-quality SE ghosts which could provide efficient protection for humans and animals. This paper describes a novel and commonly used method to produce high-quality BGs on a large scale for the first time.

## 1. Introduction

Bacterial ghosts (BGs) are hollow bacterial cell envelopes originated from Gram-negative bacteria maintaining the intact surface structure such as outer membrane proteins, adhesins, lipopolysaccharides (LPS), and peptidoglycan in the native states [1], which exhibit strong immunogenicity without detectable infectivity [2]. Recently, the wild applications of BGs have been intensively explored. On one hand, the multi-antigenic structure of BGs can effectively stimulate cellular and humoral immunity to prevent pathogenic bacterial infection [3] and serve as a new type of vaccine [4]. On the other hand, BGs have been shown to be effective biological carriers [5] to deliver drugs [6] or antigens [7] to antigen-presenting cells (APCs) such as B cells, dendritic cells (DCs), and macrophages [8].

BGs have a wide application prospect in health medicine and great research value. Therefore, to meet the increasing requirements of BGs, it is necessary to develop methods to produce BGs on a larger scale. One of the widely used BG production methods is the use of lysis protein E, which forms transmembrane tunnels connecting the inner membrane and outer membrane in the vicinity of bacterial division sites [9]. The transmembrane tunnels are 40 to 200 nm wide in diameter [10] and can efflux the cytoplasm and generate BGs with a surface structure morphologically identical to that of the living cells [1]. Lysis protein E is 91 amino acids long [10] and plays an important role in the lysis of *Escherichia coli* (*E. coli*) [11]. Lysis protein E consists of three functional domains [12], namely, a 10-amino acid-long signal peptide at the N-terminus, a transmembrane domain (TM Domain) with the structure of an alpha helix, and a hydrophilic C-terminal domain necessary for cell lysis but with no homology to any known protein domains. The overall lysis process was defined as a three-phase model: (1) integration of protein E into the inner membrane with the C-terminal domain facing the cytoplasm; (2) C-terminal domain translocated to the periplasmic space under the control of a conformation change in protein E and subsequently oligomerized to target the adhesion sites via lateral diffusion; (3) exposition of the C-terminal domain to the outer cell surface and complete fusion of the inner and outer membranes [13]. In summary, the transmembrane channel was formed by the polymerized lysis protein E at the cell division proximity connecting the cell inner and outer membranes. BGs were formed when the cytoplasmic contents were expelled from the cell bodies by osmotic pressure between the cytoplasm and the surrounding environment [14].

Currently, BGs are generally produced through inducible expression of protein E under the control of a temperature-inducible expression vector such as pBV220 without the addition of other chemicals [15]. However, this method has low yields of BGs [16]. The lysis efficiency mediated by protein E was reported to be cell density- and growth phase-dependent, where the optimal lysis growth phase was mid-log phase (OD_600_ = 0.2–0.6) [17]. This requirement led to a low yield of BGs and limited the applications of BGs. To overcome this challenge, Yu et al. [18] obtained a phage ΦX174 protein E mutant mE with an improved lysis efficiency using the gene-shuffling technique, which allowed a lysis OD_600_ as high as 1.1, with a lysis efficiency of up to 99.999%. In addition, Zhu et al. [16] found that co-expression of an A-base deletion mutant mE and *Staphylococcus aureus* nuclease A (SNA) further increased the working cell density to an OD_600_ as high as 1.2, with a lysis efficiency of about 99.9995%. However, the BG yield was still not sufficient for large-scale preparation. Alternatively to the commonly used method involving protein E, chemical treatment was another widely used method for BG production [19]. Some chemical agents such as NaOH, SDS, and H_2_O_2_ were applied to pathogens such as Gram-positive bacteria to produce BGs for vaccines [20]. However, chemical-induced methods might destroy some relevant antigenic components on the surface of the bacteria and thus reduce the desired immunogenicity in comparison to BGs prepared by protein E-based methods. 

Up to now, numerous bacteria besides the model strain *E. coli* have been successfully used to produce BGs, for example, *Salmonella enteritidis* [21], *Vibrio parahaemolyticus* [22], *Pasteurella multocida* [23], and *Bordetella bronchiseptica* [24]. *Salmonella enterica serovar Enteritidis* (*S. enterica*, SE) is a facultative anaerobic Gram-negative bacterium which frequently causes diarrhea and systemic infections of human and animals [25]. Poultry products are routinely found to be contaminated with avian *Salmonella enterica* subsp. enterica serovars [26]. Chetan et al. [27] prepared an SE ghost vaccine via a temperature-controlled expression system which can provide efficient protection against natural SE pathogens. The Lon protease encoded by the *lon* gene played an important role in the early infection and colonization of SE [28]. Therefore, Won et al. [29] evaluated the ability of an attenuated SE*Δlon* ghost vaccine to defend SE infections in chickens. The results showed that the knockout of the *lon* gene had no effect on the growth of SE, while the SE*Δlon* ghost demonstrated a good immune protection effect. Nevertheless, the problem of the low-yield production of SE ghosts still remains unsolved. Therefore, it is necessary to develop a more efficient method for the preparation of SE ghost vaccines on a large scale. 

This report is focused on improving the bacterial cell lysis efficiency mediated by protein E through genetic engineering to increase the yield of BGs. The wild-type lysis gene E (E_W_) from phage ΦX174 was synthesized in plasmid pUC57 according to its sequence from NCBI. On the basis of lysis gene E_W_, we screened for mutations that can increase the cell lysis efficiency through random gene mutation and identified that substitution of arginine at position 89 into glutamine demonstrated the highest lysis efficiency, which we named E_M_. Based on this mutant E_M_ (R89Q), several other mutants were constructed. Furthermore, a pLysS plasmid was introduced to the lysis system, which contains a T7 lysozyme coding gene. T7 lysozyme is a natural inhibitor of T7 RNA polymerase (T7 RNAP), which has little effect on bacterial growth or host gene expression [30]. T7 lysozyme can be used to reduce the expression level of T7 RNAP, which is crucial for the toxic proteins controlled by T7 RNAP systems, for example, pET plasmids. O’Mahony and colleagues found that the low expression level of T7 lysozyme in cells can significantly improve the production of recombinant protein porcine somatotropin by 40% [31]. In this study, the pLysS plasmid combined with a T7 expression system using the recombinant plasmid pET28a-E enabled bacterial lysis at an OD_600_ as high as 2.0. For the first time, we report an efficient method to prepare *E. coli* and SE BGs based on a T7 RNAP-based expression system. 

## 2. Results

### 2.1. Lysis Activity of Protein E_W_ and E_M_ with Plasmid pLysS in E. coli BL21(DE3)

**Figure 1:**
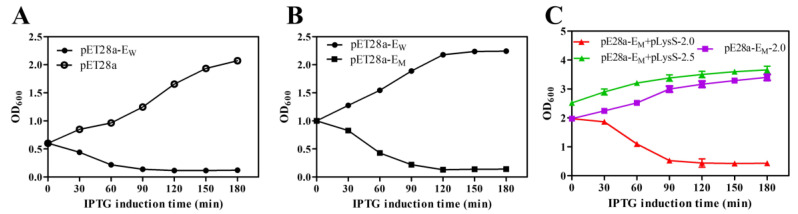
The growth and lysis curves of *E. coli* BL21(DE3) containing different lysis plasmids. The OD_600_ of bacteria cells was measured to assess the growth and lysis of bacteria (**A**–**C**). The OD_600_ values at different times are presented in the format of mean ± standard deviation (S.D). The induction concentrations of OD_600_ were 0.6 (**A**), 1.0 (**B**), and 2.0 or 2.5 (**C**). An amount of 0.1 mM IPTG was added to the cultures to induce the expression of proteins E_W_ and E_M_ at point 0.

To compare the lysis activity of lysis proteins E_W_ and E_M_, the OD_600_ value was used to evaluate the growth and lysis of bacteria. For lysis protein E_W_, the OD_600_ values of *E. coli* BL21(DE3) decreased rapidly after IPTG induction and continuously decreased for 3 h. Bacteria containing pET28a-E_W_ showed high lysis efficiency upon lysis induction at an OD_600_ value of 0.6 or lower (Figure 1A). However, protein E_W_ showed little lysis activity if compared with the mutant protein E_M_ (Figure 1B) when the OD_600_ value was increased up to 1.0, indicating that the lysis activity of the R89Q mutation improved lysis activity in later bacterial growth phases. Based on protein E_M_, several other amino acid mutations were constructed (Supplementary Figure S1). Nevertheless, these mutations did not significantly further increase the lysis activity if compared to E_M_ (data not shown). The induction time point for bacterial lysis could be increased to an OD_600_ of, at most, 2.0, thus improving the lysis efficiency of protein E and increasing the yield of BGs. However, induction at an OD_600_ of 2.5 did not further result in bacterial lysis (Figure 1C). As shown in Table 1, the lysis activity of E_M_ was improved, which reached nearly 100% when induced at an OD_600_ of 2.0. Although the initial viable bacteria concentrations differed, the cell surviving levels after two hours of protein E expression dropped to similar levels. Therefore, a significant increase in the production of BGs was achieved.

### 2.2. Morphological Observation of BGs by SEM and TEM

**Figure 2:**
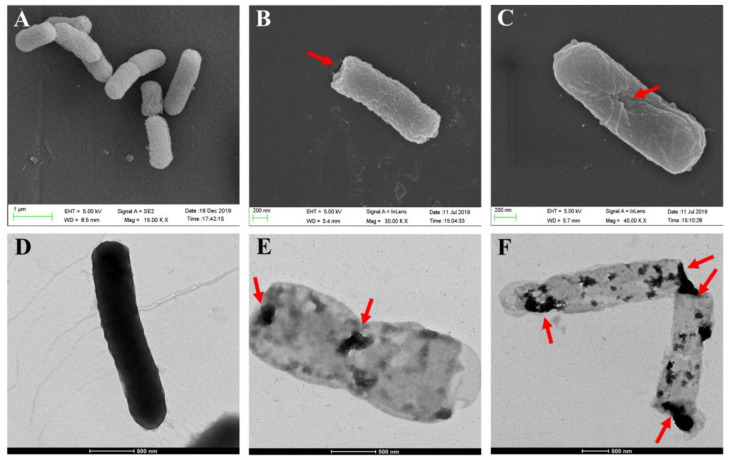
SEM and TEM analysis of *E. coli* ghosts. The untreated bacterial cells under SEM as a control (**A**). The *E. coli* ghosts with the lysis tunnels located at the poles (**B**) or at the equator (**C**) of the bacteria. The untreated *E. coli* cell, which was full of cytoplasm under TEM (**D**). The informed BGs under TEM, which contained a small amount of cytoplasm and were relatively lighter than the intact cells under TEM (**E**,**F**). The red arrow indicates the transmembrane tunnels.

The morphological features of the surface structures and internal structural features of the *E. coli* ghosts were examined by SEM and TEM. The SEM images showed that the overall morphology of the formed BGs did not differ much from the intact bacteria cells (Figure 2A), except for the lysis tunnels either at the poles or division zones of the bacteria (Figure 2B,C). As shown in Figure 2D, the color of the untreated bacteria cells was darker than that of BGs (Figure 2E,F), which represented cell envelopes devoid of an internal cytoplasm. The nucleic acid content in the BGs was greatly reduced after IPTG induction, compared with intact cells before IPTG induction (Supplementary Figure S2).

### 2.3. Immunofluorescence Microscopy and Western Blot Analysis

**Figure 3:**
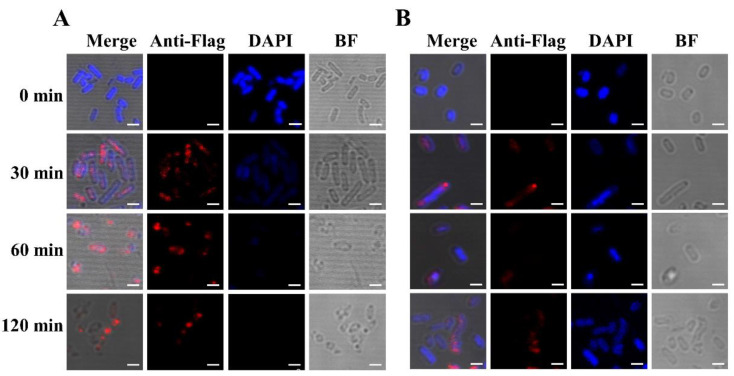
Immunofluorescence staining of lysis protein E_M_-Flag. BL21(DE3) bacterial cells containing pLysS and pET28a-E_M_-Flag (**A**) and BL21(DE3) bacterial cells only containing pET28a-E_M_-Flag (**B**) were induced by IPTG, and cells were collected at 0 min, 30 min, 60 min, and 120 min. In these bacteria cells, nucleic acid was non-specifically bound and labeled by the DPAI blue stain. The Flag tag at the C-terminus of lysis protein E_M_ was specifically recognized and bound by the mouse anti-Flag tag of the primary antibody. The Alexa Fluor^®^ 647 (red)-conjugated goat anti-mouse secondary antibody was specifically bound to the primary antibody. Bar: 2.0 µm.

A Flag tag was fused to the C-terminal end of E_M_ for detection of the lysis protein in vivo. The cells could then be stained by a Flag tag-specific antibody containing a fluorescent dye. Bacterial cells of *E. coli* BL21(DE3) containing plasmids pET28a-E_M_-Flag and pLysS were induced at an OD_600_ of 2.0. As a control, *E. coli* BL21(DE3) only containing plasmid pET28a-E_M_-Flag was also induced at an OD_600_ of 2.0. Samples were collected after induction and analyzed by fluorescence microscopy (Figure 3). Blue DAPI staining was evenly distributed throughout the cytoplasm of the bacteria cells at the beginning of induction (Figure 3A), and no red strain was observed in this group. At prolonged induction, the specific red fluorescence signal appeared, indicating the expression of lysis protein E_M_. Conversely, the blue DAPI signal gradually decreased, indicating that nucleic acid and other cytoplasmic substances were discharged through the lysis tunnel. It is worth noting that in some bacteria, the red fluorescent signal was regularly distributed at the poles or equator of the bacteria, potentially indicating the formation of E_M_-mediated lysis pores. 

**Figure 4:**
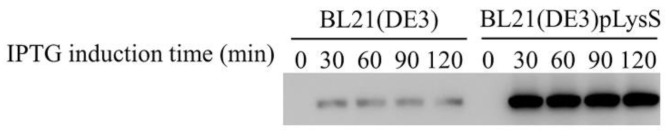
The expression levels of protein E_M_-Flag in the presence or absence of pLysS plasmid at different induction times were detected by Western blot. Time of 0 min was used as a control group that added no IPTG, and 30, 60, 90, and 120 refer to the IPTG induction times (min).

In the absence of the pLysS plasmid, a similar red fluorescent signal was detectable at the cell poles 30 min after induction (Figure 3B), while the blue DAPI stain was more persistent throughout the experiment. In accordance, Western blot analysis of the samples revealed significant differences in the expression levels of protein E_M_-Flag in the presence or absence of the pLysS plasmid (Figure 4). According to the results of immunofluorescence and Western blotting, the expression level of lysis protein E_M_-Flag was lower in the absence of the pLysS plasmid at an OD_600_ of 2.0. 

### 2.4. Construction of an Engineered SE (∆lon) Strain Expressing T7 RNAP and Being Suitable for BG Formation

**Figure 5:**
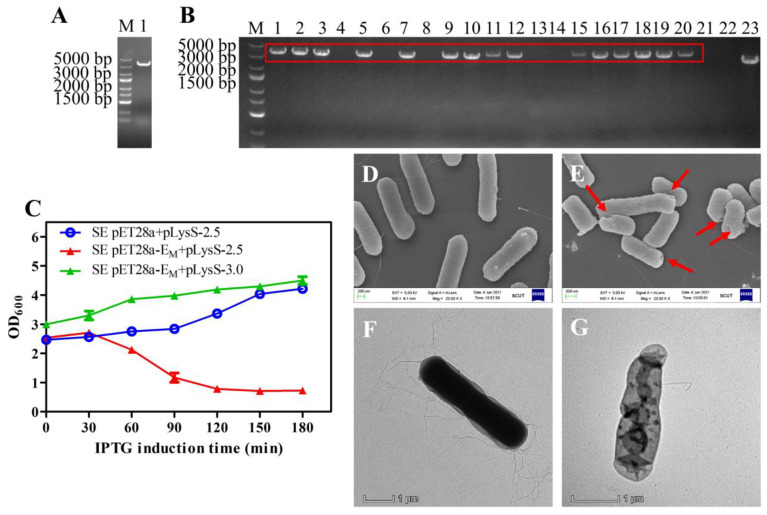
Gel electrophoresis analysis for PCR production of T7 RNAP connected with 50-bp homology arms (**A**). Gel electrophoresis analysis for SE and SE engineered strains (**B**). PCR productions of SE engineered strains (lanes 1, 2, 3, 5, 7, 9, 10, 11, 12, 15, 16, 17, 18, 19, 20) and SE wild type (lane 23) with primer 3 and primer 4 were analyzed by gel electrophoresis, where positive bands (3580 bp) are marked in a red box, and the size of the control band was 2740 bp. The lysis curves of the SE engineered strains containing pET28a-E_M_ and pLysS at late-log phase were analyzed. The OD_600_ values at different times are presented in the format of mean ± S.D (**C**). An amount of 0.1 mM IPTG was added to the cultures at time point 0, the same as in *E. coli*. SEM and TEM analysis of SE engineered strains before and after lysis was carried out (**D**–**G**). Untreated control SE cells (**D**) and the SE ghosts (**E**) were observed by SEM. The red arrows indicate the lysis transmembrane tunnels. Untreated control SE cells (**F**) and the SE ghost (**G**) were observed by TEM.

The linear fragment flanked by both 50-bp homology arms targeting the *lon* gene was generated by PCR using P1 and P2 as primers and the genomic DNA of BL21(DE3) as a template. As a result, a 3306-bp linear fragment was amplified and identified by 1.5% agar electrophoresis analysis (Figure 5A). The purified 3306-bp PCR product was transformed into the recombinant SE strain harboring the pKD46 plasmid, and colonies on the agar plates were identified by PCR using P3 and P4 primers. Bands of approximately 3580 bp were visualized in positive strains, and a band of approximately 2740 bp from wild-type SE was detected as a control, indicating that the *lon* gene was deleted and the T7 RNAP gene was inserted into the SE/pKD46 bacteria (Figure 5B). For further confirmation, the PCR product was sent for sequencing (data not shown).

### 2.5. Growth, Lysis, and Characterization of SE Ghosts

Lysis kinetics experiments showed that pET28a-E_M_ in combination with pLysS had high lysis activity in the engineered SE strains, similar to *E. coli* BL21(DE3). Effectively, lysis occurred after induction at OD_600_ values as high as 2.5, and the OD_600_ decreased rapidly within the first 2 h (Figure 5C). SEM images showed that the SE BGs retained their basic cell morphology, and the lysis tunnels were at the poles of bacteria cells (Figure 5E). Accordingly, TEM images verified the loss of cytoplasmic content from SE BGs compared with the intact cell (Figure 5F,G).

## 3. Discussion

Vaccines on the market are mainly divided into inactivated vaccines and live attenuated vaccines. Inactivated vaccines that induce a humoral immunity response have irreversible changes in their surface antigen structure, resulting in reduced immunogenicity [32]. Live attenuated vaccines that induce cellular and humoral immunity have potential hazards of virulence recovery of attenuated strains [33]. As a new strategy, bacterial ghost vaccines retain the benefits of inactivated vaccines and live attenuated vaccines while avoiding the disadvantages such as a weaker immune response, insecurity, and inconvenient transportation and storage [34]. Therefore, BGs have been widely used as multivalent vaccines [29] and DNA vaccine carriers [35] in stock farming and the aquaculture industry. BGs were also considered to be excellent and promising adjuvant systems [36]. Since the BG formation mechanism by protein E-mediated lysis has not been clarified yet, scientists cannot effectively solve the bottleneck problems such as the low lysis efficiency in the fermentation process, which severely limits BG production on a large scale. Therefore, it is urgent to improve the lysis efficiency and increase the yield of BGs.

To improve the lysis efficiency and the yield of BGs, mutants of protein E were introduced into this study. In this lysis system, the mutant protein E_M_ showed higher lysis activity than protein E_W_. This increased lysis activity may be due to the change in charge and polarity at the C-terminal of lysis protein E_M_. To illustrate this hypothesis, more experimental verifications are required. Immunofluorescence results show that part of protein E_M_ was regularly distributed in the middle or the poles of host bacteria, which was related to the formation of lysis tunnels. Therefore, the results are consistent with the hypothesis that the lysis process may be correlated with the division of bacteria [37]. 

To further improve the yield of BGs, our study introduced the pLysS plasmid developed by Studier and colleagues, which strictly controlled the expression of target genes [38]. The TM Domain of lysis protein E, a membrane protein, is composed of an alpha helix. With a small amount of T7 lysozyme, the expression of protein E_M_-Flag was significantly increased, which was proved by the results of immunofluorescence and Western blotting. Therefore, the high expression of lysis protein E_M_-Flag may lead to the increase in the lysis efficiency and the yield of BGs. 

In order to test the scope of our new technology, the T7 RNAP SE strain was successfully constructed by simplified λ Red homologous recombination technology, followed by testing and validating its effectiveness. The T7 RNAP expression system has a strong promoter induced by IPTG, which results in a high expression level of protein E_M_. These results indicate that this efficient method was feasible not only in *E. coli* BL21(DE3) but also in other T7 expression host bacteria. In this study, we optimized and explored the optimal OD_600_ value in *E. coli* and SE. The results show that the optimal OD_600_ value of *E. coli* was 2.0, and the optimal OD_600_ value of SE was 2.5. The cell lysis mechanism mediated by protein E is reported to be related to cell division, the kinetics of which is species-specific. In addition, different host bacteria usually have different division mechanisms, for example, binary fission, budding, and fragmentation, which present different-sized cell–cell interfaces. Therefore, the optimal protein induction OD_600_ value is species-specific, which needs to be optimized for each bacterium.

In conclusion, the lysis system with mutant E_M_ and pLysS has great advantages for large-scale production of BGs. The current strategy for the expression of mutant E_M_ and pLysS may provide a potential new method of obtaining efficient and high-yield BGs in the future.

## 4. Materials and Methods

### 4.1. Organism, Plasmids, and Culture Conditions

The strains, plasmids, and parts of primers used in this research are listed in Table 2. LB (Luria-Bertani) medium used in this study is one of the most common media for bacterial growth, and its rich nutrients and well-balanced components allow bacteria to grow steadily at 37 °C [39]. Appropriate antibiotics such as 30 μg/mL chloramphenicol (Cm^+^), 50 μg/mL kanamycin (Kam^+^), and 100 μg/mL ampicillin (Amp^+^) were added to culture media.

### 4.2. Protein E Plasmid Construction 

The wild-type bacteriophage ΦX174 lysis gene E (E_W_) was synthesized in plasmid pUC57 by the Beijing Genomics Institute according to its sequence from NCBI (GeneID: 2546400). On the basis of lysis gene E_W_, several other mutants including E_M_ were obtained by random gene mutation, which were cloned in plasmid pET28a by restriction-free cloning technology (RF cloning) [40]. Primers used for plasmid construction are listed in Supplementary Table S1.

### 4.3. Construction of SE with Lon Deletion and T7 RNAP Insertion Mutation

The λ Red homologous recombination system was simplified to generate a *lon* deletion and T7 RNAP insertion mutation of SE. In brief, primers P1 and P2 containing 50-nt homology arms targeting the *lon* gene and the genome of BL21(DE3) as a PCR template were used to amplify a 3306-bp linear PCR product. The resulting PCR product was purified by DNA Gel Extraction Kit (Sangon Biotech, Guangzhou, China). SE competent cells were prepared by us, and the pKD46 plasmid was transformed into SE competent cells by electroporation under the conditions of 200 Ω, 25 µF, and 2000 V. An SE recombination strain containing the pKD46 plasmid was selected and put into LB liquid medium with 100 µg/mL Amp^+^ at 30 °C. When the OD_600_ value reached 0.2–0.3, L-arabinose (L-ara) with a final concentration of 20 mM was added to bacterial cells until the OD_600_ value reached 0.6. Then, the cells were prepared into electrocompetent cells followed by transformation with approximately 100 ng of the purified PCR product. An SE recombinant inbred strain with T7 RNAP gene insertion and a *lon* gene deletion named SE/*Δlon::*T7 RNAP/pKD46 were identified by PCR using primers P3 and P4 according to the nucleotide sequence of SE available in NCBI (NZ_CP012347.1). The positive recombinant strain was cultured in LB broth at 42 °C for 16 h to remove the pKD46 plasmid. 

### 4.4. Induction Expression of Lysis Genes 

The pET28a plasmids containing different types of mutant lysis gene E were transformed into BL21(DE3) competent cells. Plasmids pET28a-E_M_ and pLysS were transformed into BL21(DE3) and SE/*Δlon::*T7 RNAP competent cells, respectively. When these recombinant strains were cultured to different OD_600_ values, 0.1 mM IPTG was added to induce the expression of the lysis genes. The growth and lysis of *E. coli* were estimated by OD_600_, and the living cell density was estimated by serial dilution and plate count methods to infer the lysis efficiency, which was calculated by the following formula: lysis efficiency = (1- post-lysis CFU/pre-lysis CFU) × 100%. At the same time, the cell density of the engineered SE strains was estimated by OD_600_. All experiments were conducted with three biological replicates. The genomic DNA from the bacteria before and after lysis was extracted by Rapid Bacterial Genomic DNA Isolation Kit (Sangon Biotech, Guangzhou, China) and analyzed by 1% agarose gel electrophoresis. Samples were collected every 30 min during the IPTG induction process. Cells were pelleted by centrifugation at 4500 rpm for 15 min and washed with PBS twice to remove the growth medium. Pellets were then immediately fixed for electron microscopy studies and immunofluorescence microscopy studies or stored at −20 °C for further Western blot analysis.

### 4.5. Scanning Electron Microscopy (SEM) 

The OD_600_ values of the above strains reached the minimum after 2 h of IPTG induction. The bacteria cells and BGs were collected and centrifuged at 4500 rpm for 15 min. Cells were resuspended in 10 mL 2.5% glutaraldehyde special electron microscopic fixative and fixed at 4 °C for 8 h. After fixation, cells were washed with deionized water 3 times and eventually resuspended in 1 mL deionized water. Samples were transferred to a dry coverslip and embedded in the filter paper. Subsequently, samples were dehydrated with a graded series (70%, 85%, 95%) of ethanol in sequence for 15 min each time and finally soaked in 100% ethanol for 15 min (this step was repeated 3 times). Following the final dehydration, samples were dried with liquid CO_2_ at the critical point using an Autosamdri-815. After drying, samples were taken out and coated by a gold sputter coater. Finally, the SEM images were acquired using HitachiS-500 SEM.

### 4.6. Transmission Electron Microscopy (TEM) 

The TEM sample acquisition was the same as that of SEM. A small amount of the washed bacteria was dipped and placed on the prepared carbon film copper mesh for 5–10 min at room temperature. About 100 µL of 3% phosphotungstic acid stain solution was added on the carbon-coated copper mesh with bacteria using a pipette. The unbound dye solution was washed off by adding deionized water on the carbon film copper net. This operation was repeated twice. Finally, the samples were observed under a transmission electron microscope (ThermoFisher scientific, Talos L120C, Waltham, MA, USA).

### 4.7. Immunofluorescence Microscopy 

This sample acquisition was roughly the same as that of SEM and TEM. *E. coli* BL21(DE3) bacteria cells induced by IPTG for 0 min, 30 min, 60 min, and 120 min were collected and centrifuged at 4500 rpm for 15 min [41]. Several buffers were prepared for further use. The fixation buffer was at the final concentration of 0.01% glutaraldehyde for electron microscopy, 2.4% paraformaldehyde, and 30 mM Na_3_PO_4_. Lysozyme was added into the GTE buffer (10 mM EDTA, 20 mM Tris-HCl, and 50 mM glucose, adjusted to pH 7.4) at a final concentration of 2 mg/mL to increase the permeability of the cell membrane.

Immunofluorescence used in this study was performed based on the previous method with slight modifications [42]. The *E. coli* bacterial pellet was washed with PBS 3 times to remove the remaining LB medium. Samples were first fixed in the fixation buffer for 15 min at room temperature and then stayed on the ice for 35 min. Cells were washed with PBS 5 times and resuspended in 200 µL GTE buffer. An amount of 20 µL of cell suspension was put on a glass-bottom cell culture dish. In order to fix the cells on the cell culture dish, 100 µL of methanol at −20 °C was dripped onto the cells and soaked for 5 min. Afterward, 100 µL of acetone at −20 °C was dripped onto the cells and soaked for 1 min. After the fixing step, cells were washed with PBS buffer 5 times. After blocking with 200 µL blocking buffer at room temperature for 1 h, cells were washed with PBS 6 times, followed by incubation with 200 µL monoclonal ANTI-FLAG^®^ M2 antibody (Sigma, St. Louis, MO, USA) at 1:500 in PBS as the primary antibody at 4 °C overnight. The next day, samples were incubated with 150 µL goat anti-mouse IgG H&L-Alexa Fluor^®^ 647 (Abcam, Cambridge, MA, UK) at 1:500 in PBS as the secondary antibody in the dark for 1.5 h at room temperature. After washing 5 times, samples were incubated with the nuclear stain 4′, 6′-diamidino-2-phenylindole (DAPI; Sigma, St. Louis, MO, USA) at a final concentration of 25 µg/mL for 15 min in the dark to identify the distribution of DNA in the cytoplasmic space. The cells were washed with PBS 5 times and observed by the fluorescence microscopy, using a Leica TCS SP5 with a 100× oil immersion objective (Leica, Wetzlar, Germany).

### 4.8. Western Blot Analysis

Western blot analysis in this study was carried out based on an established procedure with slight modifications [43]. Briefly, 10 µL protein samples of *E. coli* BL21(DE3) was separated by 16% tricine-SDS-PAGE. Proteins were blotted onto a nitrocellulose membrane (0.2 µm, GE Healthcare Bio-Sciences, Pittsburgh, PA, USA) using the Bio-Rad Transblot Turbo system at 80 V for 1 h. After incubating with the blocking buffer (5% (*w*/*v*) skimmed milk powder in PBS) for 1 h, the membrane was incubated with monoclonal ANTI-FLAG^®^ M2 antibody produced in mouse (Sigma, St. Louis, MO, USA) at 1:3000 in PBS at 4 °C overnight. The next day, the membrane was incubated with HRP-conjugated Goat Anti-Mouse IgG H&L (Abcam, Cambridge, MA, UK) at 1:3000 in PBS at room temperature for 1 h. Finally, the Super Signal West Pico Chemiluminescent Substrate (ThermoFisher scientific, Waltham, MA, USA) was used to visualize the bands on the membrane.

### 4.9. Statistical Analysis

All statistical analyses were performed with GraphPad Prism 5 software. Data were expressed in the format of mean ± S.D.

## Figures and Tables

**Table 1:** The OD_600_ and CFU/mL of *E. coli* bacteria cells containing pET28a-E_M_ and pLysS.

0 min		120 min		Lysis Efficiency (%)
OD_600_ Value	CFU/mL	OD_600_ Value	CFU/mL	
0.41	1.06 × 10^8^	0.11	2.65 × 10^3^	99.998
0.98	1.76 × 10^9^	0.22	2.33 × 10^4^	99.999
1.99	3.60 × 10^10^	0.43	4.97 × 10^4^	Nearly 100%

Note: the data are presented in the format of mean values.

**Table 2:** Strains, plasmids, and primers used in this study.

Strains Plasmids Primers	Description	Source
Strains		
DH5α	Host cells for plasmid amplification	Our lab
BL21(DE3)	Host cells for protein expression	Our lab
SE	Wild type of S*almonella enterica* subsp*. enterica* serovar *Pullorum str.* ATCC 9120	Our lab
SE*Δlon::*T7 RNAP	Deletion of *lon* and insertion of T7 RNAP in SE	This study
**Plasmids**		
pUC57-E_W_	Template of lysis gene E_W_	BGI
pET28a-E_W_	Lysis plasmid used in this study	This study
PET28a-E_M_	Lysis plasmid used in this study	This study
pLysS	Assisted lysis plasmid	Our lab
pKD46	Plasmid for λ Red homologous recombination	Our lab
**Primers**		
P1	ggtatggagcacagctatactatctgattacctggcggacactaaactaaTTTACACTTTATGCTTCCGG	
P2	cgaaatagcctgccagccctgtttttattagcgctatttgcgcgaggtcaTTACGCGAACGCGAAGTCC	
P3	GCAGGCTTCTGGCGAATAATT	
P4	CGCACCTGAATCCTTCGAAGTA	

Lowercase letters indicate the 50-nt homology arms targeting the *lon* gene.

## Data Availability

Detailed data is available upon request.

## Supplementary Materials

The following are available online at https://www.mdpi.com/article/10.3390/toxins13060420/s1, Figure S1: Alignment of lysis protein E and its mutants, Figure S2: Gel electrophoresis results of the genomic DNA in *E. coli* BL21(DE3) before lysis (lane 1) and after lysis (lane 2), Table S1: Parts of the primers used in this study.
