# Supplementary material for: A Novel and Efficient High-Yield Method for Preparing Bacterial Ghosts
Source: Toxins (Basel). 2021 Jun 13;13(6):420. doi: 10.3390/toxins13060420 (PMC8231862; doi:10.3390/toxins13060420)
Supplement: Supplementary file 1 [file toxins-13-00420-s001.zip › toxins-1253858-supplementary.pdf]

Supplementary Materials: A Novel High-Efficient Method for Preparing Bacterial Ghosts in Large Quantities

Yi Ma, Liu Cui, Meng Wang, Qiuli Sun, Kaisheng Liu and Jufang Wang

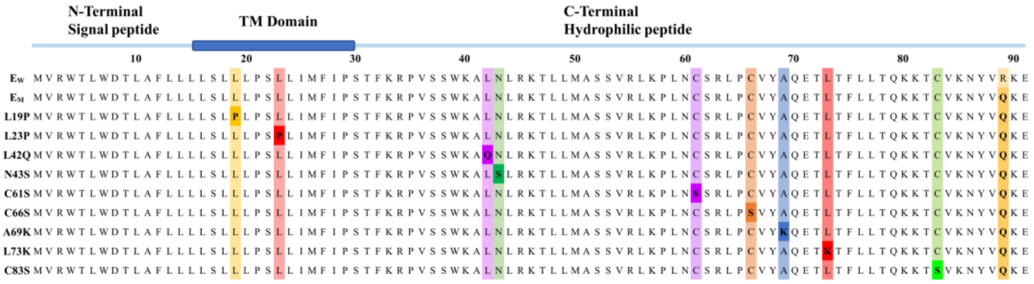

Figure S1. Alignment of lysis protein E and its mutants were listed. Ew referred to wild-type lyase, and EM was the mutation of arginine at position 89 to glutamine. The mutation sites were marked in color. Transmembrane domain (TM Domain) from the 15th to 30th was labeled.

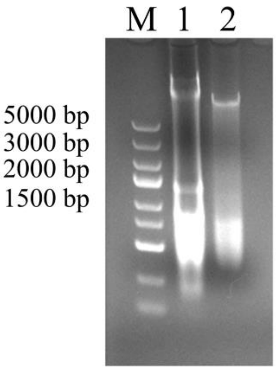

Figure S2. The genomic DNA in *E. coli* BL21 (DE3) before lysis (lane 1) and after lysis (lane 2) were analyzed by gel electrophoresis.

Table S1. Part of the primers used in this study.

Primers	Sequence (5′–3′)
F-E_W	TTTAAGAAGGAGATATACCATGGATGGTACGCTGGACTTTGTGG
R-E_W	CGGATCTCAGTGGTGGTGGTGGTGGTGCTCTTTCTGGACATAATTCTTC
F-E_M	TTTAAGAAGGAGATATACCATGG ATGGTGCGTTGGACTTTATGG
R-E_M	CGGATCTCAGTGGTGGTGGTGGTGGTGCTCTTTCTGGACATAATTCTTCAC
$F_{M/L19P}$	TTAGTCTGCCATTGCCATCATTGTTAATTATGTTCATCCCGAGCAC
$R_{M/L19P}$	AATGATGGCAATGGCAGACTAAACACTTGCTTTTCTTTTGCTGC
$F_{M/L23P}$	TCTTGCCATCACCATTAATTATGTTCATCCCGAGCACCTT
$R_{M/L23P}$	AATTAATGGTGATGGCAAGAGCAGACTAAGCAGCAAAAGAA
$F_{M/L42Q}$	AAGCACAGAACCTTCGTAAAACGCTGCTCATGGCGTCTT
$R_{M/L42Q}$	TTTACGAAGGTTCTGTGCTTTCCATGAGCTTACAGGTCTCTT
$F_{M/N43S}$	CTTAGTCTTCGTAAAACGCTGCTCATGGCGTCTTCGGTT
$R_{M/N43S}$	AGCGTTTTACGAAGACTAAGTGCTTTCCATGAGCTTACAGG
$F_{M/C61S}$	GCTTAAAACCTCTGAATTCTTCCCGGTTACCGTGCGTT
$R_{M/C61S}$	AGAATTCAGAGGTTTTAAGCGAACCGAAGACGCCATGA
$F_{M/C66S}$	ATTGTTCCCGGTTACCGTCTGTTTACGCCCAAGAAACATT
$R_{M/C66S}$	AGACGGTAACCGGGAACAATTCAGAGGTTTTAAGCGAACC
$F_{M/A69K}$	GCGTTTACAAGCAAGAAACATTAACATTCTTACTGACGCAGAAG
$R_{M/A69K}$	TGTTTCTTGCTTGTAAACGCACGGTAACCGGGAACAATT
$F_{M/L73K}$	GAAACAAAGACATTCTTACTGACGCAGAAGAAGACCTGCGT
$R_{M/L73K}$	AGTAAGAATGTCTTTGTTTCTTGGGCGTAAACGCACGGTA
$F_{M/C83S}$	ACCTCTGTGAAGAATTATGTCCAGAAAGAGCACCACCAC
$R_{M/C83S}$	ACATAATTCTTCACAGAGGTCTTCTTCTGCGTCAGTAAGAATG
